# Liver Transplantation in PNPO Deficiency: Management Challenges and Biological Lessons

**DOI:** 10.1002/jmd2.70067

**Published:** 2026-01-11

**Authors:** Richard Webster, Bindu Parayil Sankaran, Sushil Bandodkar, Michael Stormon, Gordon Thomas, Albert Shun, David Geoffrey Bowen, Timothy Fielder, Peter Barclay, Youssef Khalil, Philippa Mills, Peter Clayton, Kaustuv Bhattacharya

**Affiliations:** ^1^ T.Y. Nelson Department of Neurology and Neurosurgery Children's Hospital at Westmead Sydney Australia; ^2^ Department of Paediatrics Liverpool Hospital Liverpool Australia; ^3^ Department of Clinical Biochemistry Children's Hospital at Westmead Sydney Australia; ^4^ Department of Gastroenterology Children's Hospital at Westmead Sydney Australia; ^5^ Department of Surgery Children's Hospital at Westmead Sydney Australia; ^6^ Faculty of Medicine and Health The University of Sydney Sydney Australia; ^7^ AW Morrow Gastroenterology and Liver Centre Royal Prince Alfred Hospital Sydney Australia; ^8^ Tissue Pathology and Diagnostic Oncology Royal Prince Alfred Hospital Sydney Australia; ^9^ Pharmacy Department Children's Hospital at Westmead Sydney Australia; ^10^ Great Ormond Street Institute of Child Health University College London London UK; ^11^ Genetic Metabolic Disorders Service Sydney Children's Hospital Network Sydney Australia; ^12^ School of Medicine University of New South Wales Sydney Australia

**Keywords:** cirrhosis, hepatocellular carcinoma, liver transplantation, PLP associated hepatotoxicity, PNPO deficiency, vitamin B6

## Abstract

Pyridox(am)ine 5′ Phosphate Oxidase deficiency (PNPO) presents with refractory epilepsy responsive to treatment with pyridoxal 5′ phosphate (PLP) or pyridoxine. A 15‐year‐old boy with PNPO deficiency and cirrhosis underwent orthotopic liver transplantation for hepatocellular carcinoma without extra‐hepatic disease. Pre‐transplant, the boy was cognitively normal with well controlled epilepsy on PLP 50 mg/kg/day. Continuous EEG monitoring was used pre‐operatively and post‐operatively to identify encephalopathy resulting from PLP deficiency. B6 vitamers (pyridoxine [PN], pyridoxamine [PM], pyridoxal [PL] and phosphorylated forms [PNP, PMP, PLP]) were assayed at times of encephalopathy (symptoms and/or EEG) and for pharmacokinetics. Doses of PLP were titrated to prevent encephalopathy and limit side effects. The intraoperative/immediate postoperative periods were managed with intravenous PLP at a dose which could be reduced to 7.8 mg/kg/day before encephalopathy recurred. Post‐transplant, transition to oral PLP (100 mg/kg/day) led to fulminant hepatic impairment, which improved when IV dosing resumed. Subsequent transition to full oral PLP dosing took 9 months with a final dose of 24 mg/kg/day oral PLP. PLP showed dose dependent hepatotoxicity with associated rises in alpha‐fetoprotein levels. Gradual PLP dose changes and frequent oral dosing minimised encephalopathy episodes and hepato‐toxicity. Six years post‐transplant, liver biopsy showed moderate portal fibrosis (Ishak fibrosis stage 2/6, LAFSc score 3/9). Encephalopathy/seizures were associated with lower plasma PL concentrations (40 times above physiological levels); but high plasma PLP concentrations did not prevent encephalopathy. Despite liver transplantation, requirements for supraphysiologic doses of PLP continued, suggesting impaired neuronal PLP salvage is the major factor determining PLP requirements in PNPO deficiency.

## Take Home Message

In an adolescent with PNPO deficiency, PLP treatment was hepatotoxic and was associated with hepatocellular carcinoma. Liver transplantation did not significantly reduce requirements for high dose pyridoxal 5′ phosphate (PLP) treatment, suggesting that impairments in intraneuronal PLP recycling are the major reason that high doses of PLP are required to prevent encephalopathy. Ongoing hepatotoxicity post‐transplant resulting from treatment with PLP was associated with gradually progressive hepatic fibrosis.

## 1 Introduction

Pyridox(am)ine 5′ Phosphate Oxidase (PNPO) deficiency is an autosomal recessive disorder of vitamin B6 metabolism caused by mutations in the PNPO gene [1]. It usually presents with refractory neonatal seizures responsive to treatment with pyridoxal 5′ phosphate (PLP) or pyridoxine (PL) [2].

We have previously reported a child with PNPO deficiency who developed cirrhosis of the liver at the age of 2 years [3]. He presented in the new‐born period with intractable seizures refractory to anticonvulsants that responded to PLP at 24 days of age. Further evaluation identified heterozygous mutations in the *PNPO* gene (*PNPO* c.98A>T, c.246delT) [4]. During early infancy he had recurrent episodes of encephalopathy which were treated with escalation of PLP doses to 100 mg/kg/day. Abnormal liver function tests and early cirrhosis (on liver biopsy) were first noted at 2 years of age. His liver function tests improved concomitant with reducing the PLP dosage to 50 mg/kg/day and using shorter dosing intervals (4th hourly).

For the next 12 years his seizures remained well controlled, and he continued to have normal development. However, he had ongoing evidence of cirrhosis on liver biopsy with persistently elevated transaminases (AST and ALT approximately three times above the upper limit of normal) and an elevated alpha‐fetoprotein (αFP—twice the upper limit of normal). At 13 years and 8 months, he was found to have changes suggesting hepatocellular carcinoma (HCC) on his hepatic MRI. HCC was confirmed on liver biopsy. No extrahepatic disease was identified and he was referred for liver transplantation.

Orthotopic liver transplantation, originally developed as a therapeutic intervention for terminally ill patients with primary liver disease, has now evolved into a potential therapeutic option for a growing number of metabolic disorders [5, 6]. In monogenic disorders, it acts either by substituting an injured liver or by supplying a tissue which can replace a mutant protein [7]. Though the primary indication for liver transplant in our patient was treatment of hepatocellular carcinoma, one hypothesis was that a normal liver (with normal PNPO function) would allow hepatic production of PLP, reducing the requirements for PLP doses or allowing management with potentially less toxic B6 vitamers (pyridoxine, pyridoxamine).

This report summarises the challenges and lessons learnt from managing this patient post liver transplant. In particular, we describe the use of parenteral PLP and dose‐dependent hepatotoxicity of oral PLP. Post‐dose B6 vitamer levels enabled examination of the pharmacokinetics of oral, parenteral and nasal administration of PLP. This scenario is previously unreported and provides insight into the management of future patients with PNPO deficiency should they need liver transplantation.

## 2 Methods

### 2.1 Ethics Statement

The study was approved by the ethics committee of the Sydney Children's Hospital Network (Project No. CCR2020/29). The patient and his parents gave written informed consent for publication of the details.

### 2.2 Clinical Data

The relevant clinical data were retrieved from medical records. Given that there were no guidelines on the dosage of PLP post‐transplant or for dosing delivered by an intravenous or nasal route, management was driven by the patient's clinical response. The dose of PLP was titrated to administer the minimal dose of PLP required to prevent episodes of encephalopathy and to limit PLP‐associated hepatotoxicity. Because management was by a multi‐disciplinary team in response to acute neurological and hepatic changes, a predefined protocol could not be followed.

### 2.3 PLP Deficiency Symptoms

Symptoms and signs of encephalopathy resulting from cerebral PLP deficiency documented prior to transplant were used to identify episodes of encephalopathy post‐transplant. The first symptoms the patient reported were typically feeling unwell and anxious; then a range of other visual, autonomic, sensory, cognitive, autonomic and motor symptoms would develop. Symptoms/signs reported were having sore eyes, visual disturbance (e.g., seeing colours, spots and sometimes rainbow‐like colours), abnormal eye movements (staring, darting eye movements), distress, disorientation, sudden unexplained mood change, altered consciousness, malaise, vomiting, loss of balance and if not treated rapidly with PLP, seizures. We refer to these episodes as PLP deficiency symptoms.

### 2.4 EEG Monitoring

Given the non‐specific nature of the early symptoms of PLP deficiency (particularly post‐transplant) and the need to identify PLP deficiency at times when the patient was sedated, continuous EEG/video EEG monitoring was performed using the 10/20 system. This was commenced during the perioperative period and instituted whenever changes were made in the doses or in the mode of administration of PLP. A total of 4599 h of EEG monitoring was performed. Details regarding the EEG findings associated with PLP deficiency in this boy will be presented elsewhere.

### 2.5 PLP Administration

Continuous infusions of intravenous PLP (PLP 30 mg/mL solution Pydoxal [TaiyoPharma Japan]) were run using an infusion pump. PLP infusions were protected from light and whenever possible, were administered via opaque giving sets. Intravenous PLP boluses were also prepared using a 30 mg/mL solution, which was prepared immediately before administration and given by slow intravenous injection. Intranasal doses of PLP were prepared by drawing IV PLP (Pyridoxal 5 phosphate 30 mg/mL Pydoxal [TaiyoPharma Japan]) into a 2 mL syringe and then attaching a nasal atomiser. The patient was asked to sit back with head extended and PLP was sprayed into each nostril.

Oral PLP capsules with strengths ranging from 15 to 400 mg were prepared by the Hospital Pharmacy as required. Pharmaceutical grade PLP powder from either Medisca Australia or PCCA Australia was used to prepare capsule batches. PLP was also administered rectally by dissolving PLP in a small volume of water and then inserting a small rectal catheter and injecting the solution into the rectum. Pharmaceutical grade pyridoxamine dihydrochloride (Henan Tianfu Chemical Company) was purchased and 50 mg Pyridoxamine dihydrochloride capsules were prepared locally.

### 2.6 B6 Vitamer Assays

B6 vitamer levels were assayed to better understand the pharmacokinetics of vitamin B6 administration to establish dosing and periodicity. Fresh plasma samples were collected, protected from the light using aluminium foil and were transferred immediately to the laboratory for processing.

Six different vitamers of vitamin B6 in plasma (pyridoxal [PL], pyridoxamine [PM], pyridoxine [PN], pyridoxal 5′‐phosphate [PLP], pyridoxamine 5′‐phosphate [PMP] and pyridoxine 5′‐phosphate [PNP]) plus its breakdown product, 4‐pyridoxic acid (PA), were simultaneously measured using a modified isotope dilution Liquid Chromatography Mass Spectrometric (LC MS/MS) analytical method previously described [8]

LC–MS/MS was performed using a Waters Acquity I Class UPLC system linked to a Waters TQ‐XS mass spectrometer. A Waters Acquity UPLC HSS T3 analytical column (2.1 × 100 mm; 1.8 μm P/N 188003539) was used in conjunction with mobile phases A consisting of 3.7% acetic acid and 0.04% heptafluorobutyric acid (HFBA) in MilliQ water and mobile phase B consisting of 100% methanol, the pump flow rate being 0.4 mL/min and the column temperature set at 40°C.

All B6 vitamers, the breakdown product 4‐pyridoxic acid and their deuterated internal standards were detected in positive ion mode and differentiated using multiple reaction monitoring mode (MRM) based on mass to charge ratios and their retention times.

EDTA plasma samples stored protected from light at −80°C were treated with equal volumes of 0.3 N Trichloroacetic acid (TCA) containing deuterated internal standards, vortexed and allowed to stand on ice for 60 min. Six microliters of the deproteinised [9] supernatant obtained after centrifuging the plasma ‐TCA mixture was injected onto the LCMS/MS system.

Calibration curves were constructed using ratios of the responses of deuterated internal standards and the corresponding non‐labelled vitamers (concentrations ranging from 12 to 3000 nmol/L) to quantify all B6 vitamers (except pyridoxine 5′‐phosphate due to unavailability of a reference standard) and 4‐pyridoxic acid. In‐house tri‐level spiked plasma quality controls were prepared and used for each analytical batch.

To guide the re‐introduction of oral PLP, levels of B6 vitamers were measured after differing doses of IV and oral LP were administered. Blood was drawn at regular intervals and B6 vitamer levels were plotted. Blood levels of B6 vitamers at times when the patient had symptoms of PLP deficiency were compared with levels without deficiency using Student's *t*‐test. Reference ranges (RR) for B6 vitamer levels in plasma and CSF used previously published data [8, 9, 10].

## 3 Results

### 3.1 Liver Transplantation

A liver transplant using a whole graft from a deceased donor was performed at the age of 13 years and 11 months. Standard immunosuppression was commenced post operatively. Explant histology showed micronodular cirrhosis with a 2 cm well differentiated HCC along with several additional low grade dysplastic nodules. The course post liver transplantation was complicated by an episode of acute rejection a week after transplant and the development of a biliary stricture which initially was stented before a choledocho‐duodenostomy 5 months post‐transplant.

### 3.2 Intravenous PLP Dosing Post Liver Transplantation

Prior to liver transplantation the patient was taking a stable dose of PLP 50 mg/kg/day divided across four hourly doses with a larger night‐time dose and no overnight dose. During and immediately after liver transplantation, PLP was administered intravenously by continuous infusion. The PLP infusion rate was initiated at 1.1 mg/kg/h (26.4 mg/kg/day) and was able to be reduced to 0.35 mg/kg/h (8.5 mg/kg/day) before symptoms of PLP deficiency emerged. In order to transition from PLP infusion and to provide a more physiological pattern of delivery of PLP, IV four hourly bolus doses were then initiated at a total dose of 13 mg/kg/day and reduced to 7.8 mg/kg/day.

### 3.3 Acute Hepatotoxicity Associated With High Dose Oral PLP

**FIGURE 1:**
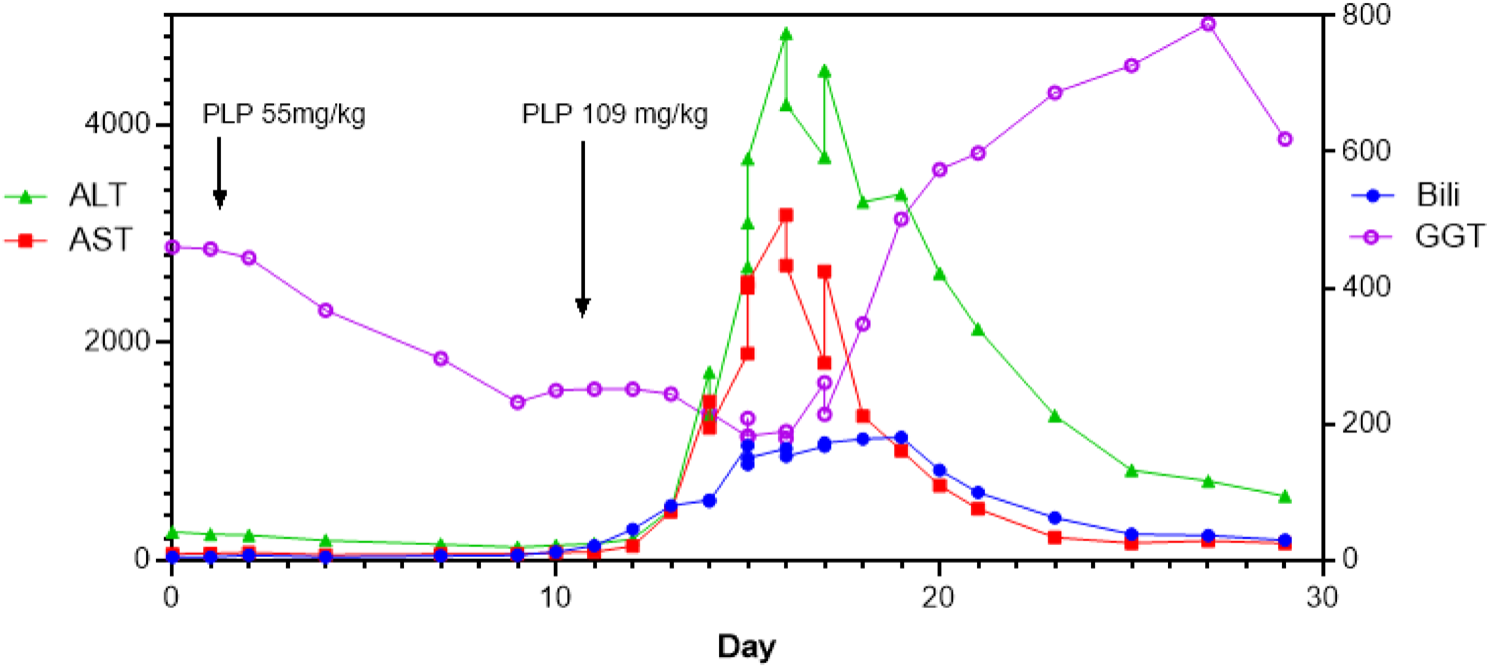
Liver function deterioration while on high dose oral PLP. This figure shows the severe hepatocellular injury and hepatic dysfunction that occurred with a rapid transition from IV PLP to high dose oral PLP. Oral PLP was initiated twenty days after transplant at the pre‐transplant dose of 2.8 g/day (55 mg/kg/day)—day 1 in graph above and then because of recurrent episodes of encephalopathy was increased to 5.6 g/day (109 mg/kg/day) on day 11. IV PLP was recommenced on day 12 at a dose of 600 mg/day and oral PLP was ceased. Liver biopsy showed extensive hepatocyte necrosis, without evidence of rejection. ALT, alanine transaminase in U/l (RR: 0–39 U/L); AST, aspartate transaminase in U/L (RR: 0–49 U/L); Bili, bilirubin in μmol/L (RR: < 10 μmol/L); GGT, gammaglutamyl transferase in U/L (RR: 0–45 U/L).

Two weeks post‐transplant, oral PLP was introduced at the pre‐transplant dose of 55 mg/kg/day in combination with IV PLP. IV PLP was then gradually withdrawn. Following this there were frequent episodes suggesting PLP deficiency (clinical and EEG) and so the oral dose of PLP was rapidly escalated to 5.6 g/day (109 mg/kg/day) over the next 10 days. On this dose of PLP the patient became nauseous and there was a rapid and severe deterioration in his liver function (Figure 1). Other than changes in the mode of delivery of PLP and the dose of PLP no other cause was identified for this episode. Extremely elevated PL levels (peaking at 93020 nmol/L [reference range (RR) 5–18 nmol/L]) were measured at the time of the acute deterioration whereas PLP levels were consistent with those seen at other times when treated with oral PLP (peak PLP 3180 nmol/L [RR 46–321 nmol/L]). A liver biopsy performed at this time showed extensive hepatocyte necrosis without evidence of rejection. The liver dysfunction resolved with re‐institution of IV PLP.

### 3.4 Transition From Intravenous to Oral Dosing of PLP

Rapid decreases in oral PLP dosage resulted in episodes of encephalopathy at doses which were subsequently tolerated if the dose was reduced very gradually (over weeks). The transition from IV PLP to oral PLP was associated with frequent episodes of hepatic dysfunction (predominantly elevations of AST/ALT and increases in αFP) which were often associated with anorexia. Liver function tests reliably improved with reductions in oral PLP dosage and resolved with the re‐institution of IV PLP treatment (which was able to be administered at a considerably lower dose). Shorter dosing intervals (3rd hourly) were eventually used which allowed a lower total daily dose of PLP to be tolerated.

Overall, it took 22 months to establish the patient on a dose of PLP which minimised both hepatotoxicity and gastrointestinal side effects (nausea, vomiting, weight loss) and controlled episodes of encephalopathy associated with PLP deficiency. The final treatment dose was 24 mg/kg/day given in three hourly doses with a larger dose at 22:00 and then no dosing overnight followed by the first morning dose at 07:00. This daily dose of PLP left little leeway should a dose of PLP be delayed or omitted.

### 3.5 Evidence of Chronic Hepatotoxicity Associated With PLP

Mild elevation of transaminases persisted 6 years post‐transplant (AST and ALT approximately 1.5 times above the upper limit of reference range), with persisting elevation of alpha‐fetoprotein (αFP—approximately 1.5 times the upper limit of reference range) without evidence of recurrent hepatocellular carcinoma on repeated imaging.

Serial liver biopsies following the episode of acute hepatic necrosis showed hepatocyte recovery but persisting mild fibrosis. A liver biopsy performed 3.5 years post‐transplant showed evidence of mild focal lobular inflammation with focal hepatocyte necrosis and mild portal fibrosis (Ishak fibrosis stage 1/6; LAFSc score 1/9) as well as mild inflammatory changes related to rejection. Liver biopsy performed 6 years post‐transplant showed moderate portal fibrosis with early fibrous and mild pericellular fibrosis (Ishak fibrosis stage 2/6, LAFSc score 3/9), with mild inflammatory rejection‐related changes.

### 3.6 Trials of Pyridoxamine and Pyridoxine

Two trials of oral PM were undertaken to see whether this allowed reduction of the IV PLP dose. In the first trial, PM (initially at a dose of 8.2 mg/kg/day and increased to 12.3 mg/kg/day in five divided doses) was added to the patient's treatment while the IV PLP bolus doses were gradually reduced from 8.4 to 6 mg/kg/day. Recurrent episodes of encephalopathy occurred which persisted despite increasing the IV PLP dose to 7.8 mg/kg/day. A further trial where a lower dose of oral PM (2.5 mg/kg/day) was added while the patient was receiving IV PLP boluses (7.8 mg/kg/day) resulted in episodes of encephalopathy which resolved when PM was ceased. A short trial of oral pyridoxine (2.5 mg/kg/day for 72 h) during an attempt to reduce an IV PLP infusion (8 mg/kg/day) did not show any added benefit and was associated with two episodes of encephalopathy. These episodes resolved when pyridoxine was ceased.

### 3.7 Trial of Rectal PLP

Prior to liver transplantation a trial of rectal PLP was initiated at a dose of 50 mg/kg/day and then increased to 56 mg/kg/day (five divided doses) following a seizure. The patient was able to be maintained on this for 48 h. A further 36‐h trial of rectal PLP (25 mg/kg/day) was undertaken post‐transplant in an attempt to use rectal absorption to partially bypass portal circulation of PLP. This was associated with recurrent episodes of PLP deficiency and a generalised tonic–clonic seizure.

### 3.8 B6 Vitamer Levels

Levels of PL, PLP, PN, PNP and PA (pyridoxic acid) were measured during IV infusion, IV bolus dosing, oral and rectal administration of PLP (Table 1). Levels of all B6 vitamers were higher while on IV infusion when compared to the trough levels taken when the same dose was administered using IV bolus administration. In turn, PLP levels were higher on IV PLP than those measured during oral administration, although PL levels were comparable, despite the oral dose being roughly double the IV dose. IV bolus PLP administration was associated with mean trough PLP/PL ratios of 2.87 ± 0.77 (mean ± 1 SD), whereas with oral administration the ratio reversed with a mean PLP/PL ratio of 0.15 ± 0.09.

**TABLE 1:** Plasma B6 vitamer levels on IV, oral and rectal PLP.

	Dose	PL^a^ nmol/L	PLP^a^ nmol/L	PM^a^ nmol/L	PN^a^ nmol/L	PA^a^ nmol/L	PLP/PL^a^
IV infusion	12 mg/kg/day	9729 (391)	41 032 (3500)	0	428 (50)	5144 (111)	4.22 (0.33)
IV bolus	12 mg/kg/day^b^	5137 (1267)	14 186 (2441)	0	305 (74.8)	3959 (110.7)	2.87 (0.77)
Oral	21 mg/kg/day^c^	5435 (883)	1627 (177)	1401 (329.5)	2415 (556)	5795 (1988)	0.31 (0.017)
Rectal	32 mg/kg/day^d^	5977 (1204)	2268 (1748)	421 (92)	1063 (279)	8452 (1994)	0.36 (0.20)

^a^ Mean (Standard Deviation).

^b^ IV PLP given as 4 hourly boluses.

^c^ Oral PLP given at three hourly intervals with a larger 22:00 dose and no dose until 07:00 the next morning. The level was the trough 07:00 level taken immediately prior to the morning dose.

^d^ Rectal PLP was given at 4 hourly intervals with a larger 22:00 dose and no overnight dose. Levels taken during a 2 day trial of rectal PLP.

**FIGURE 2:**
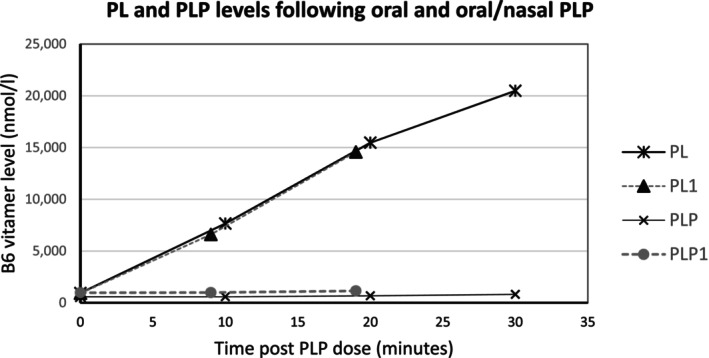
Vitamer levels following oral and oral/nasal PLP administration. This chart shows plasma levels of PL and PLP after doses of PLP of 600 mg oral (PL, PLP) and 400 mg oral and 30 mg nasal (PL1 and PLP1) were given to treat PLP deficiency symptoms. In both cases, the changes in PL and PLP following dosing were almost identical. There was a prompt rise in PL while there was little change in PLP. While not plotted on this graph, PA rose even more rapidly and peaked at a much higher level than PL in both cases (199 546 nmol/L and 48 792 nmol/L, respectively). This suggests that a large proportion of the absorbed dose was converted into PA instead of being released from the liver as either PLP or PL.

**FIGURE 3:**
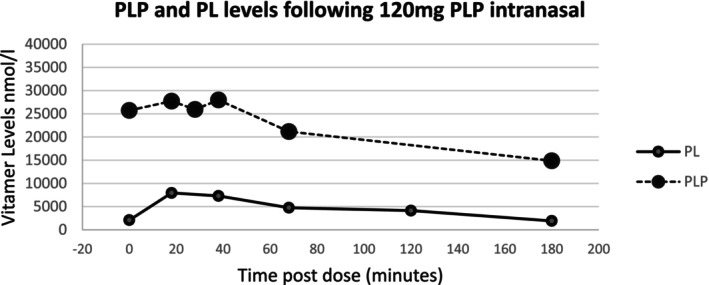
Pharmacokinetics of intranasal dosing of PLP. The chart shows levels of PL and PLP after an intranasal dose of 120 mg of the IV solution of PLP was given intranasally at time 0 after a previous dose of IV PLP had been given 4 h previously (hence the elevated baseline PLP level). PL rose from 2092 nmol/L to a peak of 7945 nmol/L within 18 min of the dose and PLP rose from 25 724 nmol/L to 27 721 nmol/L and stayed elevated at this level until 38 min post dose.

After IV PLP, PLP levels peaked 5 min after the dose whereas PL levels peaked 35–40 min after the dose. Plots of B6 vitamer levels after oral and oral combined with nasal dosing of PLP are shown in Figure 2. Serial measurements of trough B6 vitamer levels following four hourly oral dosing of PLP 200 mg (3.3 mg/kg/dose) showed that PL levels remained stable at levels above those associated with encephalopathy. Changes in B6 vitamer levels following a single intranasal dose of 120 mg PLP (intravenous solution) are shown in Figure 3. This dose was given 4 h after ceasing IV PLP. Following intranasal administration of 120 mg PLP IV solution, both plasma PL and PLP levels rose within 20 min of administration. The use of intranasal PLP was considered as a maintenance treatment and a short trial of this (120 mg four hourly for 8 h) was performed but the practicalities of the currently available formulation (IV PLP 30 mg/mL) meant that the volume needed was not well tolerated.

Table 2 shows plasma levels of B6 vitamers measured at times when well documented episodes of PLP deficiency symptoms occurred (clinical ± EEG features). Plasma levels of B6 vitamers (PL, PLP, PM, PN) measured at times of PLP deficiency when on oral or rectal treatment were significantly lower than levels at times when there was no evidence of deficiency—(PL—Deficiency 1129 ± 524 nmol/L [mean ± 1SD], No deficiency 8529 ± 4623 [*p* < 0.001]; PLP—Deficiency 760 ± 257 nmol/L, No deficiency 1328 ± 1075 [*p* < 0.05]; PM—Deficiency 245 ± 46 nmol/L, No deficiency 738 ± 326 [*p* < 0.001]; PN—Deficiency 409 ± 145 nmol/L, No deficiency 1460 ± 768 [*p* < 0.001]). All episodes of encephalopathy occurred with PL levels below 2800 nmol/L although levels below this were sometimes seen without PLP deficiency symptoms especially if PLP levels were high (e.g., while on IV PLP). Conversely, while low circulating levels of PLP were frequently associated with low PL levels, high blood levels of PLP did not always protect against encephalopathy. Two episodes of encephalopathy occurred despite very high blood levels of PLP (20 785 nmol/L and 17 772 nmol/L [N 46–321 nmol/L]) while the patient was receiving a combination of IV PLP and oral PM. While the sum of PLP and PL levels was significantly lower during episodes of encephalopathy, there were two episodes where PLP deficiency symptoms occurred (noted above) where the combined circulating level was very elevated (during PM administration).

**TABLE 2:** Plasma B6 vitamer levels during episodes of encephalopathy on oral, rectal and IV PLP.

Mode of PLP administration	PL nmol/L (5–18)^a^	PLP nmol/L (46–321)^a^	PM nmol/L (0)^a^	PN nmol/L (0–0.6)^a^	PA nmol/L (16–139)^a^
Oral	790	384	182	226	1673
Oral	915	970	310	310	2359
Oral	939	587	274	333	2415
Rectal	2225	793	224	446	3209
Rectal	1223	732	230	533	2364
Rectal	1278	1098	275	609	13 290
Median (range)	1081 (790–2225)	762 (384–1098)	252 (182–310)	389 (226–609)	2389 (1673–13 290)
IV^b^	2727	20 785	5076	121	2372
IV^b^	2318	17 772	4941	102	1998

^a^ Control range Footit et al. (8).

^b^ These episodes occurred while on a combination of therapy with IV PLP 5.8 mg/kg/day and oral PM 8 mg/kg/day (hence the elevated levels of PM).

B6 vitamers were measured in CSF while on an IV PLP infusion (720 mg/day–12 mg/kg/day) and compared with control values [9, 10]. CSF B6 vitamers were very elevated (PLP 273 nmol/L [RR 11–34 nmol/L], PL 929 nmol/L [RR 16–36 nmol/L], PN 231 nmol/L [RR < 0.03 nmol/L] and PM 575 nmol/L [RR 0.3–0.9 nmol/L])—PA, PNP and PMP were not detected. While no paired blood sample was taken at this time, blood levels taken the previous day on the same infusion rate were PLP 32 000 nmol/L and PL 9000 nmol/L, giving a plasma/CSF ratio of 117 for PLP and 9.7 for PL.

## 4 Discussion

### 4.1 Liver Transplantation Did Not Cure PNPO Dependent Epilepsy

Liver transplantation provided a unique opportunity to understand some of the factors influencing PLP metabolism in PNPO deficiency. This patient had normal hepatic PNPO function after transplantation and hence normal hepatic PLP metabolism but remained PNPO deficient in the central nervous system. If hepatic PNPO metabolism was the main driver of the need for PLP, it was hypothesised that liver transplantation would either decrease or eliminate the need for PLP or alternatively allow the administration of other less toxic B6 vitamers (e.g., pyridoxine or pyridoxamine). Neither of these hypotheses was supported. Despite liver transplantation, the patient had an ongoing need for supraphysiologic doses of PLP and had episodes of encephalopathy which occurred despite elevated plasma levels of B6 vitamers. Neither pyridoxamine nor pyridoxine administration allowed the dose of PLP to be reduced, although the trials of both vitamers were relatively short.

Prior to liver transplant the patient was receiving 50 mg/kg/day PLP and post‐transplant the PLP dosage was able to be reduced to 24 mg/kg/day. While it is possible that lower requirements for PLP resulted from liver transplantation, it seemed more likely that more aggressive titration of PLP dosage in response to hepatotoxicity allowed the dose to be minimised.

All episodes of encephalopathy post‐transplant occurred with circulating supraphysiological levels of PLP and PL. While we have no data about intraneuronal B6 vitamer levels, the CSF level of PLP and PL was approximately 10‐fold higher than control levels. Animal data show that vitamin B6 concentrations within the brain are higher than CSF levels [11] although it is unclear how this applies to humans. PNPO deficiency would not be expected to impair the delivery of PL across the blood brain barrier. As such, it seems likely that impairments in the neuronal salvage pathway for PLP [12] resulted in the persisting requirement for supraphysiological doses of PLP.

### 4.2 IV PLP Requirements Were Approximately One Third of Oral PLP Requirements

Given the need for parenteral therapy during transplant and in the post‐transplant period, IV PLP was administered either by infusion or intermittent intravenous boluses for a period of several months. IV PLP proved very effective in preventing episodes of encephalopathy. The minimum IV PLP dosage needed to avoid episodes of PLP deficiency was approximately one third of the oral dose required. PLP associated hepatotoxicity was not seen while on IV PLP (despite considerably higher PLP levels); however, the use of IV PLP was followed by a very difficult and prolonged transition from IV to oral PLP (approximately 6 months). It is not clear why this occurred. Higher intraneuronal levels of PL and PLP may have resulted in neuronal adaptation (alterations in B6 metabolism or adaptation of PLP dependent enzymes to higher levels of PLP), and these changes were slow to re‐adjust to the lower circulating B6 vitamer levels seen with oral administration.

### 4.3 Oral PLP Showed Dose Dependent Hepatoxicity Post Liver Transplant

After liver transplantation, re‐introduction of oral PLP was associated with dose dependent hepatotoxicity. The rapid introduction of high dose PLP (up to 100 mg/kg/day) was followed by severe liver dysfunction with extensive hepatocyte necrosis on liver biopsy. These changes resolved when oral therapy was stopped and IV PLP was re‐initiated at a dose more than 10‐fold lower. Subsequent episodes where liver function deteriorated when on oral PLP improved when the total dose of PLP was decreased and reliably resolved when IV PLP was re‐initiated. Liver biopsies performed post‐transplant showed evidence of ongoing hepatotoxicity with moderate fibrosis at 6 years post‐transplant (Ishak Fibrosis grade of 2/6, LAFSc score 3/9); however, well short of recurrent cirrhosis.

We and others have previously reported PLP associated hepatotoxicity [13, 14]. The cause of PLP associated hepatotoxicity is not known although PLP is an aldehyde and so is likely to be unstable and may have off‐target consequences [12, 15]. Given that hepatotoxicity occurred post‐transplant (in a liver with normal PNPO function) and reliably improved when a lower dose of oral PLP was given, it appears that this was a direct hepato‐toxic effect of oral PLP or of one of its metabolites. When oral PLP was discontinued and IV PLP was started, the resolution of hepatotoxicity was reliable and rapid. This may have been because: (a) IV PLP escaped first‐pass hepatic metabolism by being delivered outside the portal circulation, (b) the IV PLP dose required to prevent deficiency symptoms was approximately one third of the oral dose and (c) IV PLP avoided intestinal metabolism by gut microbiota and so it is possible that toxic PLP metabolites were not produced. The occurrence of hepatotoxicity in the transplanted liver indicates that the hepatotoxicity of PLP in patients with PNPO deficiency is not primarily the result of abnormal PLP metabolism due to hepatic PNPO deficiency and is consistent with other reports of PLP associated hepatotoxicity occurring in people without PNPO deficiency [14].

### 4.4 B6 Vitamer Levels Varied According to the Mode of Administration

B6 vitamer levels varied considerably according to the mode of PLP administration. With oral administration of PLP, plasma PL levels were higher than PLP levels whereas with IV administration the ratio was reversed. The relatively elevated PLP levels with IV infusion are consistent with the mode of administration. With oral dosing of PLP, elevation of plasma PL levels compared to PLP has previously been reported both in patients with PNPO deficiency and also in patients with PLP responsive epilepsy without identified genetic defects in B6 metabolism [8]. After oral dosing with PLP, the liver is considered predominantly to export PLP [9]; the finding of elevated PL has been hypothesised to be due to limitations in the capacity of albumin to bind PLP resulting in PLP metabolism by alkaline phosphatase [16]. Plasma PM and PN levels in our patient were considerably higher than those previously reported in patients without PNPO deficiency treated with a similar dose of PLP [8]. This is unexplained.

Given the few episodes of PLP deficiency symptoms where blood was collected, it is difficult to draw strong conclusions about how well blood levels of B6 vitamers predicted these episodes. Low PL levels were most strongly associated with these episodes, and all occurred with PL levels below 2800 nmol/L; however, at times similar levels were tolerated without symptoms. While on oral PLP, low plasma levels of PLP were frequently associated with encephalopathy. This was not always the case on IV PLP, and there were a couple of occasions where very high plasma levels of PLP were seen with encephalopathy, although admittedly both occurred while on PM.

It seems likely that plasma B6 vitamer levels are an imperfect marker of intraneuronal reserves of PLP. CSF levels of B6 vitamers (in particular PL and PLP) might be a better marker of intraneuronal B6 vitamer levels. Unfortunately, the need for lumbar puncture limits the practicality of using CSF levels to titrate PLP dosing. Plasma B6 vitamer levels have a role in guiding oral dosing of PLP, particularly where there are questions about the absorption or metabolism of PLP.

Trials of both PM and PN were short (all were less than a week), and in these trials, either PM or PN was added to existing dosing with PLP. These trials were terminated because of episodes of encephalopathy that occurred with doses of PLP, which had previously been tolerated. We had hoped that a transplanted liver with normal PNPO activity would allow PNP and PMP to be metabolised to PLP, potentially avoiding PLP associated hepatotoxicity. It is possible that the high intrahepatic levels of PLP needed to manage this boy inhibited PNPO and so limited the metabolism of these vitamers to PLP. Given the short duration of these trials, it is difficult to exclude the possibility that PM or PN might have a role post‐liver transplant, and it is possible that extremely gradual introduction and escalation of either PM or PN might be tolerated.

In order to decrease hepatotoxicity, doses of PLP were minimised but this increased the risk of episodes of PLP deficiency. As such it became very important to have rescue medications available to prevent the progression of these episodes to seizures. Large oral/oral and nasal doses of PLP resulted in a rapid rise in PL levels which were well above the levels associated with PLP deficiency symptoms. Given the effectiveness of intranasal dosing of midazolam for status epilepticus, intranasal PLP was also trialed to see whether it was effective in managing PLP deficiency symptoms. Off‐label use of intranasal PLP (120 mg of IV solution) given at times of encephalopathy proved effective in addressing episodes of encephalopathy. B6 vitamer levels collected after a single intranasal dose of PLP administration showed a rise in blood PL but also a rise in PLP (not seen with oral dosing), suggesting that PLP may have been directly absorbed across the nasal mucosa. Given the need to rapidly increase circulating B6 vitamer levels during episodes of PLP deficient encephalopathy/seizures, intranasal PLP may have a role in treating these episodes while limiting the dose that needs to be given.

This study reports the challenges associated with liver transplantation in a patient with PNPO deficiency. However, given the variable clinical phenotypes seen in PNPO deficiency (e.g., PN responsiveness versus PLP responsiveness), it is unclear how well the findings of this study can be applied to other patients with PNPO deficiency who might need a liver transplant. Moreover, this patient's epilepsy was treated very aggressively with high doses of PLP for the first 14 years of his life and so he was protected from secondary epileptogenesis due to processes such as gliosis/neuronal damage because of untreated PLP deficiency/seizures. The management of patients with secondary epileptogenesis is likely to be considerably more difficult post liver transplant as treatment with PLP is unlikely to stop all epileptic activity. While this study used levels of B6 vitamers to guide PLP dosing post‐transplant, the so‐far unique situation of having a patient with PNPO deficiency with normal hepatic PNPO activity means that the B6 vitamer levels we measured are unlikely to reflect those seen in patients with PNPO deficiency who have not undergone liver transplantation.

## 5 Conclusion

Liver transplantation in this adolescent with hepatocellular carcinoma was curative and he remains disease free. The post‐transplant management proved to be more challenging than anticipated. Oral treatment with PLP was eventually able to be re‐established after a very gradual transition from IV dosing to oral dosing. The prolonged use of IV PLP proved very effective in preventing episodes of encephalopathy associated with PLP deficiency and minimised PLP associated hepatotoxicity. Unfortunately, liver transplantation did not remove the need for supraphysiological doses of PLP. Post‐transplant there was evidence of ongoing dose‐related PLP hepatotoxicity with gradually progressive hepatic fibrosis 6 years post‐transplant although this was well short of cirrhosis. Given emerging reports of cirrhosis in people with PNPO deficiency, it is possible that other patients may need liver transplantation. Hopefully, this report will provide guidance for the management of other patients with PNPO deficiency where liver transplantation is being considered.

## Funding

The authors have nothing to report.

## Conflicts of Interest

The authors declare no conflicts of interest.

## Data Availability

The data that support the findings of this study are available on request from the corresponding author. The data are not publicly available due to privacy or ethical restrictions.
